# Risk of *Ixodes ricinus* Bites in a Population of Forestry Workers in an Endemic Region in France

**DOI:** 10.3390/pathogens13080696

**Published:** 2024-08-16

**Authors:** Antoine Grillon, Erik Sauleau, Nathalie Boulanger

**Affiliations:** 1 UR3073, PHAVI, Groupe Borrelia, Université de Strasbourg, F-67000 Strasbourg, France; a.grillon@unistra.fr; 2 Centre National de Référence Borrelia, CHRU Strasbourg, F-67200 Strasbourg, France; 3 ICube UMR 7357—Laboratoire des Sciences de L’ingénieur, de L’informatique et de L’imagerie, Laboratoire, CS 10413, F-67412 Illkirch Cedex, France; erik.sauleau@chru-strasbourg.fr

**Keywords:** *Ixodes ricinus*, risk factors, tick bite, forest ecosystem, tick control

## Abstract

The progressing worldwide increases in tick occurrence and tick-borne diseases calls for the development of new prevention strategies to reduce their impact on human and animal health. Defining the risk of exposure to tick bites is therefore essential. Forestry workers are at high risk of tick bites. We set up an explorative study among forestry workers in the Alsace region in eastern France to measure the different factors affecting the risk of *Ixodes ricinus* tick bites during their activities in forests. For one year, forestry workers recorded the presence of ticks on their clothes and tick bites every time they were working in teams in different forest ecosystems. Questions about the prevention measures they followed were also noted. Among the 32 participants, we were able to differentiate between groups having a high, neutral, or low risk of being bitten. The median tick bite number per year was 4 (0–8). We tried to identify individual as well as environmental factors affecting the risk of tick bites. Factors influencing the risk were the seasonal peak of tick activity in May and June, the time of exposure, and the forest ecosystems visited during the year. Additional factors potentially affecting the risk were also identified.

## 1. Introduction

Ticks are the number one vector of diseases affecting animals and humans [1]. For several decades, ticks habitats have been expanding due to climate change and socioeconomic changes [2]. There are about 900 species of ticks, which are divided into two main families: hard ticks or *Ixodidae* and soft ticks or *Argasidae* [3]. The hard tick *Ixodes ricinus* is the most abundant species in Europe [4]. It finds favorable environments in the vegetation and leaf litter of deciduous or mixed forests and in coniferous forests with at least 80% relative humidity [4]. *Ixodes ricinus* is a generalist tick, feeding on more than 300 wild animal species [1,5]. Wild animals such as rodents and deer are essential for their survival [6,7]. The hematophagous behavior of all *Ixodes* tick stages, i.e., larvae, nymphs, and adult females, ensures the effective transmission of different potentially pathogenic microorganisms to humans: bacteria (*Borrelia burgdorferi* sensu lato complex, *Anaplasma phagocytophilum*), viruses (tick-borne encephalitis (TBE) virus), and parasites (*Babesia* spp.) [8]. Seroprevalence studies of Lyme borreliosis and TBE virus showed that forestry workers are particularly exposed to tick bites [9,10].

We conducted an exploratory study to determine the potential factors affecting the risk of tick bites and TBDs. We collaborated with forestry workers from Alsace, a region in eastern France, where ticks and TBDs are endemic [11]. These workers are regularly exposed to *I. ricinus* during their activities in forested areas [4]. After a preliminary questionnaire on their field experience with ticks, we asked participants to report ticks on their clothing and tick bites every time they were in the forest, which was in groups of two to seven, every month for a year. We also collected information on the visited site and the number of hours spent in a tick ecosystem. With the data from the thirty-two initial participants of this study, we first performed a global analysis of their exposure to tick bites. We then selected a team of seven forestry workers to more specifically measure the risk of tick bites during regular hammering activity. Finally, we attempted to identify the most important risk factors influencing the risk of tick bites.

## 2. Material and Methods

### 2.1. Participant Recruitment

The participants were forestry workers from the Alsace region, including the Bas-Rhin (Lower Rhine) and Haut-Rhin (Upper Rhine) departments, an area in which Lyme borreliosis and other TBDs are endemic [11,12]. In the Bas-Rhin department, 19 subjects were identified initially, and 15 people finally participated in this study in 2015–2016. In the Haut-Rhin department, 13 subjects were identified, and 11 participated in this 2018–2019 study. Six participants discontinued their involvement in this study. Participants in both departments typically worked as teams of 2 to 7 in the same forest area, multiple times every month, mostly for 4 to 7 h each day, during the hammering period.

A questionnaire was handed out to all participants before this study to collect information on their own perception of sensitivity to tick bites, the most frequent location of tick bites, their hygiene habits, the use of prevention measures, and their history of tick bites.

They were then asked to self-report their monthly outings in groups in the forest with the detected number of ticks on their clothing and the number of tick bites on a prepared worksheet. Most participants continued their monitoring for one year as planned; however, a few individuals participated only for a few months. They were kept in the global study since the number of exposures was sufficiently high per month in these few months.

### 2.2. Statistics

Based on the participant’s self-reported data regarding the number of ticks on their clothing and tick bites, the study population was stratified into 3 groups: high, neutral, and low risk of being bitten. First, a mixed Poisson regression was constructed, explaining the number of ticks (added up for each month) for each using the duration of the outings (added up for each month, in the form of an offset to model a rate of ticks per hour of outing), the month (using dummy variables with August as the reference), and the year. To these fixed effects, a random effect specific to the subject (random intercept) was added. This last effect measured the individual deviation from an average attractiveness of all individuals (calculated as the ratio between the self-reported number of tick bites and the number of outing hours). The model for participant *i* being bitten during month *t* of year *y* a total of *n_ity_* times in a monthly total hours in the forest *d_ity_* was

$$\log\left(n_{ity}\right)=\log\left(d_{ity}\right)+\alpha_{y}+\beta_{t}+\gamma_{i}$$

Here, $\alpha_{y}$ represents the effect of the year, $\beta_{t}$ is the effect of the month, and $\gamma_{i}$ is the random effect. A K-medoids clustering (more robust than K-means) was then used to create the 3 groups according to this random effect. The attractiveness for each group was then described (mean, standard deviation, minimum, maximum, and median).

## 3. Ethics Statement

This observational “Prefortic” study was approved by the Ethics Committee of the University and Hospital of Strasbourg (reference number CE-2024-69) and registered at clinicaltrials.gov (NCT06492668), (accessed on 1 August 2024). The participants were properly informed about this study during information sessions organized by the forestry department (health and hygiene committee and physician) and gave their consent by submitting their initial questionnaire before starting the observation protocol. The participants had the right to withdraw from this study at any time. The collected survey data were pseudonymized by coding with numbers prior to analysis.

## 4. Results

Thirty-two (32) forest workers were enrolled in total in this study (Bas-Rhin department: 19, Haut-Rhin department: 13). Twenty-six (26) of these participants provided sufficient data to be included in the analysis set (Bas-Rhin: 15, Haut-Rhin: 11). The mean age of the evaluated population was 45 [range: 22–64] years (Bas-Rhin: 43 [range: 22–60] years, Haut-Rhin: 48 [range: 26–64] years). Fifteen participants (57.7%) were less than 50 years old, and eleven (42.3%) were 50 years or older (Table 1). They were all regularly exposed to tick bites during their forestry activities, most of them presenting a reaction to the tick bite within 24 h after the exposure (Table 1). Most tick bites occurred on the lower limbs, then the trunk, with fewer on the arms. In 12 cases, there was no specific site preference. Eight subjects reported earlier clinical experiences with TBDs, which are not described further in this report.

Protective measures included clothing (100%) and some additional repellents (14.5% in total). The following prevention attitudes were reported: one subject applied a commercial skin repellent (icaridine), two used essential oil, and one used a cloth repellent. Twenty-three subjects (88.5%) took at least one shower every day, and four of them twice daily. Two individuals (7.7%) took four showers per week, and one subject did not respond (Table 1). As part of the mandatory prevention for forestry workers, they always wore fully covering clothes, and some of them also wore gaiters.

**Figure 1:**
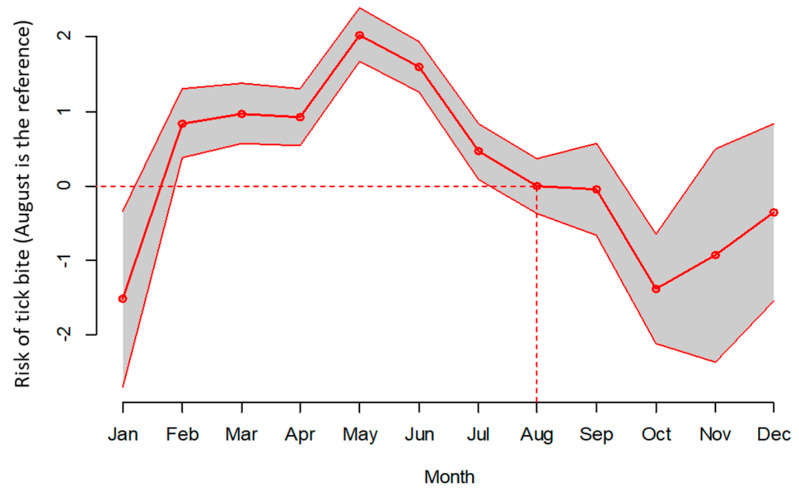
Risk of tick bites according to the month of the year, including the 2015–2016 and 2018–2019 studies, using a Poisson regression model.

The results of the overall risk analysis with the total number of exposures for all participants are presented in Figure 1. Using the observed data and using the year 2015 as reference, the tick density was found to be higher in 2016 and 2018, and tick density was about equal between 2019 and 2015. With August serving as the reference, fewer ticks were present between September and January and more were present from February to July. The fixed part of the estimated Poisson regression model confirmed these considerations according to the observation data (Figure 1). In the model, the random effects had a mean of 0.03532 (they were assumed to be normally distributed with a zero mean) and a standard deviation of 1.1533 and were estimated from −2.0730 to 2.0967.

**Figure 2:**
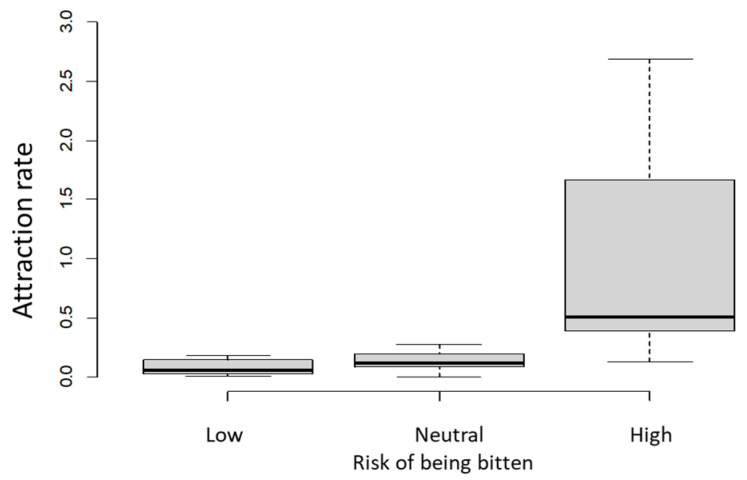
The attraction rates (number of ticks per hour of outing for each participant) for three clusters of tick bite risk, ‘low’, ‘neutral’ and ‘high’, determined using Poisson regression model.

Regarding the risk of being bitten, 9 individuals were assigned to the low-risk group (one of the three clusters according to K-medoids method), 9 were classified as neutral risk, and 13 as high risk. The attraction rates (number of ticks per hour of outing) of these clusters were not different between the neutral and ‘high’ groups (Figure 2). In those in the group with a low risk of being bitten, the mean attraction rate was 0.0837, whereas this mean was 0.1284 in the neutral-risk cluster and 1.2576 in the high-risk cluster. The clusters partially overlapped because the minimum attraction rate was 0.0099 in the low-risk group but 0.000 in the neutral-risk group. The maximum rates were 0.1782 and 0.2759 for the low and neutral groups, and the minimum in the high-risk group was 0.1290 (Figure 2).

A more detailed analysis of the risk of tick bite exposure was performed with a team of seven forestry workers who were particularly compliant with the study procedures from May 2015 to April 2016 (Table 2). The peak in tick activity was observed in May–June 2015 in the global study, as presented in Figure 1. One participant was at a low risk of tick exposure (participant #6), two had a neutral risk (participants #1 and 7), and four had a high risk (#2, 3, 4, and 5). The median tick bite number over the whole observation period (May 2015 to April 2016) was four per subject [0–8]. Participant #5, who had the highest number of tick bites, showered regularly, did not smoke, and did not use repellents. Participants #2, 3, and 4 did not use repellents and showered regularly, and one was a smoker (#3).

## 5. Discussion

In this explorative study on the exposure of forestry workers to tick bites, we selected the Alsace region, in which ticks and TBDs are endemic. This choice was supported by the existence of prior studies on the seroprevalence of Lyme and other TBDs in this population [9,10]. An epidemiological study performed in the Alsace region from 2013 to 2016 at four sites on tick density and Lyme infection prevalence in infected nymphs reported a nymph density of between 4 and 143 per 100 m^2^ and a density of *Borrelia*-infected nymphs of less than one infected nymph to more than eight per 100 m^2^ [12].

We noticed significant differences in the susceptibility to *I. ricinus* tick bites among individuals. We selected the hammering process since the forestry workers are exposed to the same risk of exposure to tick bites during this process. They move forward close to each other, one chooses a tree, and then the second measures and marks it with a hammer. The risk of tick bites is higher in some forested ecosystems than others due to their vegetation and the presence of hosts, particularly during the peak of tick activity (May–June). These preliminary data involving a small number of subjects and a limited collection of variables show that some people might be more at risk of being bitten by ticks than others. In the Poisson regression, we modeled the evolution of the bite rate as a function of ‘fixed’ variables, not dependent on each subject. A number of variables specific to the subject were also considered; they could have been used in a second stage to explain the risk: frequency of baths and showers, type of hygiene product used, use of repellent, smoking, age, and occupation. This study was carried out on a sample that was too small to significantly explain the risk associated with these variables.

The statistical Poisson model confirmed the role of month in each year and the multi-year evolution. The clustering of the participants into groups of low, neutral, and high risk of being bitten was not relevant, especially during the peak of tick activity in a forest ecosystem. Despite the limitations of this study with only 26 participants, the results of the team of seven forestry workers show that some persons are at higher risk of tick bites than others. Two people were rarely bitten over a 12-month period (no bites or one bite), some received intermediate numbers of bites (two to five), whereas some were regularly bitten (eight bites).

The risk posed by tick bites to humans was mainly based on factors like ecosystem, the density of *Ixodes* nymphs in the environment, and the duration of activity in a forest. Other factors were probably involved such as individual protective measures. A minority used skin repellents in the present study (14.5%), which is a lower percentage than in the general population in this region (24.9%) [13]. However, forestry workers always wear protective clothing, take showers after forest activities, and perform body examinations. Although forestry workers follow strict rules for prevention, the occurrences of nymphs on clothing and tick bites were not negligible. The wide distribution of nymphs in the environment and their size explain why they are the most common stage at which ticks bite humans [13,14].

The risk of tick bites is also associated with the tick’s ability to detect their hosts. Ticks possess a powerful sensory organ, Haller’s organ, which is located on the foretarsus of the first pair of legs, which is a potent chemosensation organ [15,16,17]. It detects phenols [17] and carbon dioxide [18], which are attractive to ticks. In addition, some specific bacterial volatiles have been identified as being responsible for mosquitoes’ attraction to humans, particularly *Brevibacterium epidermidis*, a commensal skin bacteria [19,20,21,22]. Although no volatiles from skin bacteria have been identified as attractants for ticks so far, the attraction of ticks to humans seems to vary, as certain individuals report more susceptibility to tick bites than others (N. Boulanger—personal survey of forestry workers).

Regular exposure to tick bites could reduce the risk of bites and TBDs [23] by exposing the host to tick saliva, which is rich in bioactive molecules [24,25,26], against which hosts produce specific antibodies and skin reactions [23,27]. This process has been shown to induce resistance to tick bites in certain laboratory animals [28] and in people in an area in which Lyme disease is endemic [23]. In our study, all except three participants reported itch after tick bite. Although speculative, the regular exposure of forestry workers to tick bites may lead to an allergic reaction, resulting in tick removal. This preliminary research needs to be taken further to identify the various factors associated with individual characteristics or specific ecosystems and thus to develop better prevention strategies.

## Figures and Tables

**Table 1:** Main characteristics of the 26 forestry workers finally enrolled in the study (Bas-Rhin and Haut-Rhin) according to questionnaire responses.

Characteristic		Subjects	
		*n*	%
Sex	Male	26	100
Age	Less than 50 years	15	57.7
	50 years or more	11	42.3
Regular exposure	4 to 7 times a month in group	26	100
Bite reaction—itch	Yes	16	61.5
	No	10	38.5
Shower frequency	Once a day	19	73
	At least twice a day	4	15.4
	Four per week	2	7.7
	ND	1	3.9
Toilet product	Gel or soap	26	100
Protective measure	Chemical repellent (icaridine)	1	3.9
	Essential oil	2	7.7
	Clothing repellent	1	3.9
	None	22	85.5
Smoker	Yes	4	15.5
	No	22	84.5
Site of tick bite	Everywhere	12	46
	Lower limbs	10	38.5
	Upper, lower limbs	4	15.5

**Table 2:** Follow up of a team of 7 participants during their outings for hammering from May 2015 to June 2016 with recording of the number of ticks on their clothing (C) or fixed on their skin (F). The number of outings per month is variable as well as the number of participants per outing (See Supplementary Data for details).

Day	2015							2016						Total of Ticks
Month	May	June	July	August	October	November	December	January	February	March	April	May	June	
Time of Exposure (h) for Each Outing	4	4 to 7	2 to 8	4	4	4 to 7	4	4 to 7	4	4 to 8	7 to 4	4 to 8	4 to 7	
Participant #	Number of ticks on clothing (C) or fixed on the skin (F)—in parentheses: number of outings													
1C	0 (5)	1 (2)	0 (6)		0 (3)	0 (4)	0 (1)	0 (2)	0 (2)	0 (5)	0 (3)			1
1F	0 (5)	0 (2)	1 (6)		0 (3)	0 (4)	0 (1)	0 (2)	0 (2)	0 (5)	0 (3)			1
2C	0 (3)	3 (5)	6 (7)	0 (5)	0 (1)	0 (1)	0 (2)	0 (2)	0 (1)	0 (3)	1 (2)	44 (3)	11 (4)	65
2F	0 (3)	3 (5)	2 (7)	0 (5)	0 (1)	0 (1)	1 (2)	0 (2)	0 (1)	1 (3)	1 (2)	7 (3)	9 (4)	24
3C	3 (2)	2 (5)	1 (4)	0 (2)	0 (3)	0 (4)	0 (2)	0 (4)	0 (3)	0 (2)	0 (3)	3 (4)	1 (5)	10
3F	0 (0)	3 (5)	2 (4)	0 (2)	0 (3)	0 (4)	0 (2)	0 (4)	0 (3)	0 (2)	0 (3)	1 (4)	1 (5)	7
4C	2 (4)	1 (3)	2 (5)	1 (3)	0 (4)	0 (2)			1 (3)	0 (2)	0 (4)		4 (3)	11
4F	2 (4)	0 (3)	2 (5)	0 (3)	0 (4)	0 (2)			0 (3)	0 (2)	0 (4)		0 (3)	4
5C	4 (5)	4 (4)	7 (8)	0 (1)	0 (3)	0 (2)	0 (2)			6 (2)	2 (4)	48 (3)	18 (5)	89
5F	2 (5)	2 (4)	3 (8)	0 (1)	1 (3)	0 (2)	0 (2)			0 (2)	0 (4)	4 (3)	5 (5)	17
6C	0 (2)	0 (7)	0 (7)	0 (3)							0 (2)	0 (3)	0 (3)	0
6F	0 (2)	0 (7)	0 (7)	0 (3)							0 (2)	0 (3)	0 (3)	0
7C	0 (3)	0 (3)	0 (6)	0 (2)	0 (2)	0 (2)	0 (2)	0 (2)	0 (1)	0 (3)		30 (2)	21 (2)	51
7F	0 (3)	0 (3)	1 (6)	1 (2)	0 (2)	0 (2)	0 (2)	0 (2)	0 (1)	0 (3)		4 (2)	6 (2)	12

“C”: ticks present on clothing; “F”: ticks fixed on skin.

## Data Availability

The raw data supporting the conclusions of this article will be made available by the authors on request.

## Supplementary Materials

The following supporting information can be downloaded at: https://www.mdpi.com/article/10.3390/pathogens13080696/s1, Table S1: Details for the outings for hammering with the date of the month, the number of hours spent in the ecosystem, number of participants per outing.
